# RNA-Binding Proteins and Their Emerging Roles in Cancer: Beyond the Tip of the Iceberg

**DOI:** 10.3390/ijms24119612

**Published:** 2023-06-01

**Authors:** John J. Murphy, Kalpana Surendranath, Radhakrishnan Kanagaraj

**Affiliations:** 1 Genome Engineering Laboratory, School of Life Sciences, University of Westminster, 115 New Cavendish Street, London W1W 6UW, UK; k.surendranath1@westminster.ac.uk; 2 School of Life Sciences, University of Bedfordshire, Park Square, Luton LU1 3JU, UK; 3 Centre for Drug Discovery and Development, Sathyabama Institute of Science and Technology, Chennai 600119, India

RNA-binding proteins (RBPs) represent a large family of proteins with an extensive array of roles that contribute to coordinating and directing multiple functions in RNA metabolism and transcription. Currently, there are upwards of 1500 RBPs, and unravelling their range of complex functions in cell biology is an ongoing research effort, where there is still much to discover. Although RBPs primarily interact with RNA in both the nucleus and cytoplasm, these interactions often have a wider influence on cell biology, including effects on genomic stability and critical cell protein levels. Recent studies have shed light on specific RBPs and their roles in cancer, but the detailed molecular mechanisms responsible for the reported roles of RBPs in tumorigenesis have hitherto been largely unexplored. This Special Issue, entitled “RNA-Binding Proteins and Their Emerging Roles in Cancer” in the International Journal of Molecular Sciences (IJMS), includes seven contributions, three original articles, and four reviews covering a wide range of highly relevant topics in RBP biology and cancer. These include cancer therapies, post-transcriptional regulation and genome integrity, non-coding RNA regulation, RNA and DNA damage responses, and R-loop biology. A summary of the contributions that make up this recently published IJMS Special Issue are provided below.

Nasiri-Aghdam et al. [1] provided an interesting, timely review on the pleiotropic CUGBP Elav-like family (CELF) of RBPs, which comprise six members of CELF1-6. These proteins are found in the nucleus and cytoplasm of cells. The major functions of these proteins include regulating mRNA alternative splicing in the nucleus and the regulation of mRNA stability, translation, and alternative polyadenylation in the cytoplasm by binding to consensus sequences in the 3′UTR regions of mRNAs. Intriguingly, almost 15% of CELF1 binding sites are found in intergenic regions, which primarily include non-coding RNAs (ncRNAs); therefore, the cross-regulation of CELF RBPs and ncRNAs and its potential importance in cancers is a focus of this review. There is currently more published information on CELF1 and CELF2 in cancers than on CELF3-6 proteins. CELF1 and CELF2 often have tumour suppressor functions, but their potential oncogenic roles in certain cancers were also highlighted [1]. In the future, the pharmacological targeting of CELF proteins may be useful in novel cancer treatments [1]. Notably, CELF1 aggregation in the perinucleolar compartment (PNC) occurs more frequently in cancer cells, suggesting that organelle-specific drug delivery and targeting could be a potential therapeutic approach for cancer treatments [1].

Another small family of RBPs that regulate alternative splicing, such as the CELF RBPs, are the muscleblind-like (MBNL) RBPs, MBNL1, MBNL2, and MBNL3. The multi-functional roles of MBNL RBPs were evidenced in a research article by Cai et al. [2]. MBNL2 has reported roles in tumorigenesis and the authors investigated the effect of MBNL2 knockdown on the transcriptome of cancer cell lines. The authors found a strong connection between MBNL2 knockdown and a reduction in cyclin-dependent kinase inhibitor 1A (p21^CDKN1A^) expression, which is mediated by the effects of MBNL2 on the modulation of p21 mRNA stability. They presented evidence that these effects are independent of the p53 protein [2]. MBNL2 knockdown also results in increased checkpoint kinase 1 (CHK1) S345 phosphorylation, which can be rescued by an overexpression of p21 [2]. Finally, the authors showed that MBNL2 depletion increased DNA-damage-induced apoptosis, while inhibiting DNA damage repair and DNA damage-induced cellular senescence [2].

The review provided by Sidali et al. [3] was concerned with eight proteins, which are all well characterised as AU-rich element RBPs (AU-RBPs) and have been reported to bind to mRNAs containing AUUUA motifs in their 3′UTR, thereafter mediating either mRNA degradation or stabilisation. These proteins mediate their post-transcriptional regulation of gene expression effects, primarily in the cell cytoplasm; however, many of these proteins can also be found in the nucleus. This review focused on canonical and emerging non-canonical functions, specifically in relation to their roles in maintaining genomic stability [3]. AU-RBPs post-transcriptionally regulate the expression of the mRNAs involved in DNA damage response and signalling, for instance, ZFP36 stabilises Claspin mRNA, which is critical for the activation of CHK1 via ATR. In response to genotoxic stress, AU-RBPs can be post-translationally modified and shuttle between the cytoplasm and nucleus to carry out specific functions. Evidence for the nuclear functions of AU-RBPs in maintaining genomic stability is sparse, but there are some interesting avenues being explored, such as the role of intronic AU-rich elements, which do appear to have a role in regulating mRNA expression via the AU-RBP HuR. In addition, emerging evidence points to a role for certain AU-RBPs, for instance AUF1, in preventing the formation of R-loops after DNA damage and inhibiting the defective DNA damage response that can promote genomic instability. The reported roles for AU-RBPs in cancer are numerous; for example, AU-RBP ZFP36L1 has been reported as a driver gene for human breast cancer.

Löblein et al. [4] produced an interesting research article concerning post-transcriptional regulator Musashi proteins (MSI-1 and MSI-2) in ovarian cancer. MSI RBPs functions have mostly been reported in stem cells and Löblein et al. [4] investigated whether these RBPs maintain the cancer stem cell characteristics in ovarian cancer. Furthermore, as MSI1 and MSI2 have a high homology and overlapping functions, they also investigated whether a dual knockdown of both of these proteins might have therapeutic potential in ovarian cancer. MSI expression in ovarian cancer is positively correlated with cancer stem cell related genes. A dual knockdown of MSI1 and MSI2 reduced cell growth and increased the sensitivity of ovarian cancer cell lines to chemotherapy, suggesting that this strategy may have therapeutic potential in this cancer.

Dolicka et al. [5] presented a review of stress granules (SGs) and their emerging roles in hepatocellular carcinoma (HCC). SGs form in cells in response to various cell stress signals and are membrane-free cytosolic compartments where mRNAs are protected and translationally silenced. Interestingly, increased SG formation is associated with several liver diseases, including HCC. Dolicka et al. [5] reviewed the current knowledge of SG formation and analysed the SGs proteome. Many proteins in SGs are also associated with HCC and include numerous RBPs and scaffold proteins, etc., which have been reported as tumour suppressors or oncogenes in different cancers. Although an oncogenic role for SGs in HCC has not yet been firmly demonstrated, the authors reviewed the emerging evidence for such a role and focused on the selected protein and nucleic acid components of SG that might have the potential to be pharmacologically targeted in future therapeutic strategies for HCC.

Uruci et al. [6] provided a comprehensive review on R-loops, which are three-stranded structures that arise when RNA/DNA hybrids form after an RNA strand hybridises to a transcribed DNA template strand, displacing the non-transcribed DNA template as a single-stranded DNA. They arise naturally in cells as a consequence of a number of physiological processes, and these R-loops, termed “scheduled” R-loops, are mostly resolved quickly. R-loops can also have pathological functions and these “unscheduled” R-loops arise as a result of cellular dysregulation. The formation of “unscheduled”, persistent and uncontrolled, R-loops can lead to DNA damage and genomic instability. This dual nature of R-loop formation was explored by Uruci et al. [6], who made compelling arguments for the physiological functions of R-loop formation, including intriguing data that were consistent with “unscheduled” R-loops having a role in DNA repair. These authors also discussed the cellular components and mechanisms for preventing and resolving/removing R-loops and provided details on specific “Chro-Mates” that function either as chromatin modifiers or chromatin remodelers, thus highlighting the importance of chromatin accessibility and condensation for the presence of RNA/DNA hybrids. Readers are encouraged to read the Taneja paper to acquire new insights into the RNA/DNA damage response and the latest developments in this new area of study.

Nascakova et al. [7] provided a timely research article elucidating the role of the homologous recombination factor Rad51 in regulating the R-loop formation in human cells. Using the U-2-OS-based cellular system with an inducible expression of the mutant RNaseH1 enzyme, which can recognize and bind but not cleave R-loops, the authors showed that the chemical inhibition of Rad51 by B02 specifically causes the formation of R-loops in the early G1 phase of the cell cycle. Furthermore, they demonstrated that the formation of B02-induced G1-specific R-loops requires the presence of RAD51 and RNA polymerase II transcription initiation. Finally, they presented evidence that B02 treatment disrupts the pre-replication complex and promotes premature origin firing and the initiation of DNA synthesis in the early G1 phase of the cell cycle. In addition, B02 treatment was shown to accumulate R-loops near rDNA transcription start sites.

A common theme that is evident in the publications in this Special Issue is that RBPs are present in both the cytoplasm and the nucleus, often shuttling between these two cell compartments and other cytosolic compartments such as stress granules and P bodies. These observations are consistent with multiple potential regulatory functions for individual RBPs in RNA transcription and metabolism. Increasing evidence is emerging that links mutations in RBPs to tumorigenesis. There is evidence that some RBPs can regulate the stability of the RNAs involved in cell cycle control and apoptosis. Additionally, certain RBPs appear to be involved in maintaining genomic stability through direct or indirect mechanisms. At least some RBPs have roles in the regulation of R-loops, which are increasingly considered to be major sources of genomic instability. A picture is emerging of a complicated interplay between the RBPs regulating multiple functions in RNA transcription and metabolism that are also crucial for maintaining genomic stability and avoiding unwanted effects on the genome, such as the generation of R-loops when RNA transcription conflicts with DNA replication. Interestingly, mounting data identify RNA/DNA hybrids as immunogenic species that abnormally accumulate in the cytoplasm following R-loop processing. Future research into the functions of RBPs in controlling these cytoplasmic RNA/DNA hybrids, the resulting innate immune activation, and its associations with many disorders, including cancer, would be intriguing.

To date, most RBPs have generally been considered as non-tractable for therapeutic purposes. Novel approaches, such as proteolysis-targeting chimera (PROTAC) strategies, may enable selected RBPs to soon become tractable. It is likely in the future that new therapeutic strategies coupled with more detailed analyses of tumour molecular signatures and the growth of personalised medicine will result in selected RBPs becoming useful therapeutic targets in cancer therapy.

As Guest Editors for this IJMS Special Issue, we are pleased to present the collection of riveting articles focused on the topic of RBPs in cancer. This collection of articles develops the idea that RBPs have significant effects on genomic stability and cellular homeostasis, which have an impact on cancer at many levels and through various pathways. We thank the authors for their excellent contributions that add fresh perspectives to this fascinating and vitally important field of genome biology.
